# Involvement of p53 Mutation and Mismatch Repair Proteins Dysregulation in NNK-Induced Malignant Transformation of Human Bronchial Epithelial Cells

**DOI:** 10.1155/2014/920275

**Published:** 2014-08-18

**Authors:** Ying Shen, Shuilian Zhang, Xiaobin Huang, Kailin Chen, Jing Shen, Zhengyang Wang

**Affiliations:** ^1^Department of Clinical Medicine, Zhejiang Medical College, Hangzhou 310053, China; ^2^Department of Pathology and Pathophysiology, Zhejiang University School of Medicine, Hangzhou 310058, China; ^3^Department of Pulmonology, Sir Run Run Shaw Hospital, Hangzhou 310016, China

## Abstract

Genome integrity is essential for normal cellular functions and cell survival. Its instability can cause genetic aberrations and is considered as a hallmark of most cancers. To investigate the carcinogenesis process induced by tobacco-specific carcinogen NNK, we studied the dynamic changes of two important protectors of genome integrity, p53 and MMR system, in malignant transformation of human bronchial epithelial cells after NNK exposure. Our results showed that the expression of MLH1, one of the important MMR proteins, was decreased early and maintained the downregulation during the transformation in a histone modification involved and DNA methylation-independent manner. Another MMR protein PMS2 also displayed a declined expression while being in a later stage of transformation. Moreover, we conducted p53 mutation analysis and revealed a mutation at codon 273 which led to the replacement of arginine by histidine. With the mutation, DNA damage-induced activation of p53 was significantly impaired. We further reintroduced the wild-type p53 into the transformed cells, and the malignant proliferation can be abrogated by inducing cell cycle arrest and apoptosis. These findings indicate that p53 and MMR system play an important role in the initiation and progression of NNK-induced transformation, and p53 could be a potential therapeutic target for tobacco-related cancers.

## 1. Introduction

As a dominant risk factor for lung cancer, cigarette smoking has attracted researchers' great attention during the past decades. Among the numerous carcinogenic compounds in cigarette smoke, the tobacco-specific nitrosamine NNK (4-(methylnitrosamino)-1-(3-pyridyl)-1-butanone) is the most potent one [1, 2]. It not only causes pulmonary adenocarcinoma in rats, mice, and hamsters, but is also one of the human carcinogens determined by the International Agency for Research on Cancer (IARC). It has been reported that genetic polymorphisms and genomic instability are important ingredients that promote early development of NNK-induced tumorigenesis [3].

The* TP53* tumor suppressor gene has been demonstrated to be at the centre of a regulatory network that guards genome integrity in the living cells. In the presence of DNA damage, p53 protein can be activated and then promotes the expression of several important genes that are involved in cell cycle arrest, DNA repair, and apoptosis. p53 mutation and dysfunction have been found in over 50% of all types of human cancers, resulting in inactivation, silence, or even dominant-negative inhibition of wild-type p53 [4]. For human lung cancer, p53 also appears to be the major target for genetic damage in smoking-induced carcinogenesis [5, 6]. Most mutational “hot spots” have been observed clustering in exon 5–8, within the DNA binding domain of p53 [7]. In the studies of NNK induced lung tumors, specific damage distribution patterns were found and factors other than NNK adduct formation may contribute to the mutagenesis of* TP53* [8, 9].

Another fundamental mechanism for maintaining genome integrity is the DNA mismatch repair (MMR) system. In the mammalian MMR system, the heterodimeric complexes MSH2-MSH3/MSH6 (MutS) recognize mispaired bases and insertion-deletion loops and then the MLH1-PMS2 complex (MutL) interacts with MutS and orchestrates downstream DNA repair events [10]. Molecular defects in MMR genes are associated with microsatellite instability (MSI), a type of genomic instability, accounting for a significant proportion of hereditary nonpolyposis colorectal carcinoma (HNPCC) and other tumors. There are also increasing evidence revealing that MSI is involved in the early lung cancer progression [11, 12]. Genetic and epigenetic alterations of MMR genes have been found in lung cancer patients and associated with tumor suppressor gene inactivation, such as* TP53 *[11, 13]. However, little is known about the involvement of MMR machinery in NNK-induced tumorigenesis.

To investigate the role of MMR system in NNK induced carcinogenesis, we analyzed the expression level of MMR proteins during NNK-induced malignant transformation of human bronchial epithelial cells. One of the important MMR proteins, MLH1, presented a significant downregulation as early as one week after NNK exposure and maintained the low expression level throughout the malignant transformation process. Further studies indicated that changes in histone modification patterns rather than DNA methylation alterations were involved in this process. We also identified a G>A mutation at codon 273 (R273H) of* TP53* gene in NNK transformed cells. With this mutation, p53 was impeded from the rapid induction and subsequent transactivation of target genes in response to DNA damage. Reintroduction of wild-type p53 into the transformed cells resulted in the proliferation inhibition, which is associated with cell cycle arrest and apoptosis.

## 2. Materials and Methods

### 2.1. Cell Culture and Chronic Carcinogen Exposures

Normal human bronchial epithelial (NHBE) cells were purchased from XiangYa Central Experiment Laboratory (Hunan, China) and cultured in RPMI-1640 medium supplemented with 10% fetal bovine serum (Invitrogen). The protocol of chronic carcinogen exposures was performed as described previously [14–16] with modifications. Exponentially growing culture of NHBE cells was treated with 2 mM NNK (Toronto Research Chemical) for 24 h as one cycle of exposure. The dose of NNK was confirmed to be nontoxic for a 24 h exposure. After treatment, the cells were detached with trypsin/EDTA (0.05% trypsin and 0.53 mM EDTA, Invitrogen) and seeded at appropriate densities for another cycle of exposure. The cells were treated for 4 or 8 cycles in total and named as NHBE-NNK4 and NHBE-NNK8, respectively. Dimethyl sulfoxide (DMSO) was used as solvent control.

### 2.2. Plasmid Construction and Transient Transfection Assays

The coding sequence of full length human* TP53* obtained by RT-PCR from HeLa cells was inserted to a pCDNA3.1 vector with a 6 × myc tag (Invitrogen). The sequence of the construct was confirmed by DNA sequencing (Invitrogen). NHBE-NNK8 cells were transfected with the myc-p53 plasmid using X-tremeGENE HP DNA transfection reagent (Roche).

### 2.3. Methyl Thiazol Tetrazolium Assay (MTT)

MTT assay was used as a proliferation assay to assess cell growth. Cells were cultured in complete medium on 24-well plates. After 24 h, 48 h, and 72 h, MTT was added. After incubating at 37°C for 3-4 h, the absorbance at 490 nm was recorded in each well using an ELISA microplate reader.

### 2.4. Anchorage-Independent Colony Formation Assay

NHBE and NHBE-NNK8 cells (4,000 cells/well) were suspended in 0.35% agarose over a 0.7% agar base in 6-well plates, covered with 2 mL complete RPMI-1640 medium. Medium was replaced every 3 days and after 2 weeks incubation at 37°C in a humidified incubator containing 5% CO_2_; colonies with more than 50 cells were counted.

### 2.5. Wound-Healing Assay

Cells were grown to 90% confluence in 6-well plates, and a scratch was made on a uniform layer of cells using a sterile micropipette tip. Cell debris was removed by washing with phosphate-buffered saline (PBS). The rates of cell growth and migration to close the wound were observed and photographed every 24 h.

### 2.6. Immunoblotting

Total cell lysates were prepared by the addition of RIPA lysis buffer (Millipore) containing proteinase inhibitors (Roche). Protein extracts were separated in SDS-PAGE and transferred to nitrocellulose membrane (Whatman). The membrane was blocked and incubated with primary antibodies including rabbit anti-p53, rabbit anti-p21, rabbit anti-myc, and mouse anti-GAPDH (Santa Cruz), rabbit anti-MSH2, rabbit anti-MSH6, rabbit anti-MLH1, and rabbit anti-PMS2 (Abcam). Membranes were then washed with tris-buffered saline (TBS) and followed by Alex 680- or IR 800-conjugated secondary antibody for Odyssey LICOR analysis (Li-COR, Bioscience).

### 2.7. Real-Time Quantitative PCR and Methylation-Specific PCR

Total RNA was isolated with RNAsio Plus reagents (TaKaRa). Real-time quantitative PCR (qPCR) was performed using SYBR Premix Ex Taq (TaKaRa) on an ABI Prism 7500 real-time PCR system (Applied Biosystems). *β*-Actin was used as loading control. Primers were as follows: MSH2, forward, 5′-ACGATGGATTTGGGTTAG-3′, reverse, 5′-CCAGGGCTTTCTGTTTAG-3′; MLH1, forward, 5′-TCCCGAAAGGAAATGACT-3′, reverse, 5′-TTGGTGGTGTTGAGAAGGT-3′; MSH6, forward, 5′-AAGGCGAAGAACCTCAAC-3′, reverse, 5′-TCCATCTTGGCCCAAACC-3′; PMS2, forward, 5′-AGCCACTGCTGGATGTTG-3′, reverse, 5′-AGTCTTTGGGCTGTGAGG-3′; *β*-actin, forward, 5′-TGGAGAAAATCTGGCACCACACC-3′, reverse, 5′-GATGGGCACAGTGTGGGTGACCC-3′; p21, forward, 5′-CACCGAGACACCACTGGAGG-3′, reverse, 5′-GAGAAGATCAGCCGGCGTTT-3′; P53R2, forward, 5′-TCTCCCTCACTGGAACAAGC-3′, reverse, 5′-ACCTGCACCTCCTGACTAAA-3′; PUMA, forward, 5′-GACCTCAACGCACAGTA-3′, reverse, 5′-CTAATTGGGCTCCATCT-3′.

The methylation status of the promoter region of human* MLH1* and* PMS2* gene was analyzed by methylation-specific polymerase chain reaction (MSP-PCR) after DNA modification with sodium bisulphite (EZ DNA Methylation Gold Kit, Zymo Research). PCR products were electrophoresed on a 3% agarose gel. For* MLH1*, primers for the unmethylated reaction were 5′-GAAGAGTGGATAGTGATTTTTAATGT-3′ and 5′-ATCTCTTCATCCCTCCCTAAAACA-3′ and for the methylated reaction were 5′-AGCGGATAGCGATTTTTAACGC-3′ and 5′-TCTTCGTCCCTCCCTAAAACG-3′ [17]. For* PMS2*, primers for the unmethylated reaction were 5′-GTAGGTGGGAAGTTTTATATGGAG-3′ and 5′-CCAATCTCCATCATAACCTCTAACA-3′ and for the methylated reaction were 5′-AGAGGCGCGTCGTTTTCGTG-3′ and 5′-CTCCGTCGTAACCTCTAACG-3′.

### 2.8. Chromatin Immunoprecipitation (ChIP) Assay

ChIP assays were performed using SimpleChIP enzymatic chromatin IP kits (Cell Signaling Technology) according to the manufacturer's protocol. Antibodies against H3K4me3, H3K9ac, and H3K9me2 (Abcam) as well as a negative control, rabbit IgG, were used for ChIP assay. Two microliters of purified DNA from cross-linked cells was used for real-time PCR. Inputs consisted of 2% chromatin before immunoprecipitation. Primers for amplification of* MLH1* promoter were as follows: forward, 5′-ACCGCTCGTAGTATTCGTGCTC-3′; reverse, 5′-GTGGATGACGCCCAAAAGAAG-3′. Primers for amplification of* PMS2* promoter were as follows: forward, 5′-GTGTTGAGT CATTTCCCACA-3′; reverse, 5′-ATCAACACTTGATAGTCTTA-3′.

### 2.9. Microsatellite Instability Analysis

MSI analysis was done by evaluating a reference panel of five mono- and dinucleotide markers (*BAT-25*,* BAT-26*,* D2S123*,* D5S346*, and* D17S250*) recommended by the National Cancer Institute [18]. The analysis was performed as previously described [19].

### 2.10. p53 Mutation Analysis by Direct Sequencing

Genomic DNA was isolated using the genomic DNA extraction kit (Axygen). p53 mutation analyses were conducted as described by Ko et al. [20].

### 2.11. Luciferase Assay

The luciferase activities were measured as described previously [21]. The cells were cotransfected with 0.5 *μ*g of firefly luciferase reporter constructs and 0.02 *μ*g of pRL-SV40 Renilla luciferase reporter plasmids (Promega, Madison, WI) and examined by a dual-luciferase reporter assay system (Promega).

### 2.12. Cell Cycle Analysis

NHBE-NNK8 cells were transfected with the empty vector or the myc-p53 plasmid for 48 hours, permeabilized by 70% ethanol at −20°C, and incubated with 50 *μ*g/mL PI and 20 units/mL RNase-A (Roche Diagnostics). The cells were analyzed by flow cytometry using Coulter EPICS XL analyser (Beckman Coulter) and FlowJo 7.6 software.

### 2.13. Statistical Analysis

All data are representative of at least three independent experiments. Statistical analysis was performed by one-way ANOVA and the results were regarded significant when *P* < 0.05. Error bars represent s.d.

## 3. Results

### 3.1. Malignant Transformation of NHBE Cells with NNK

**Figure 1:**
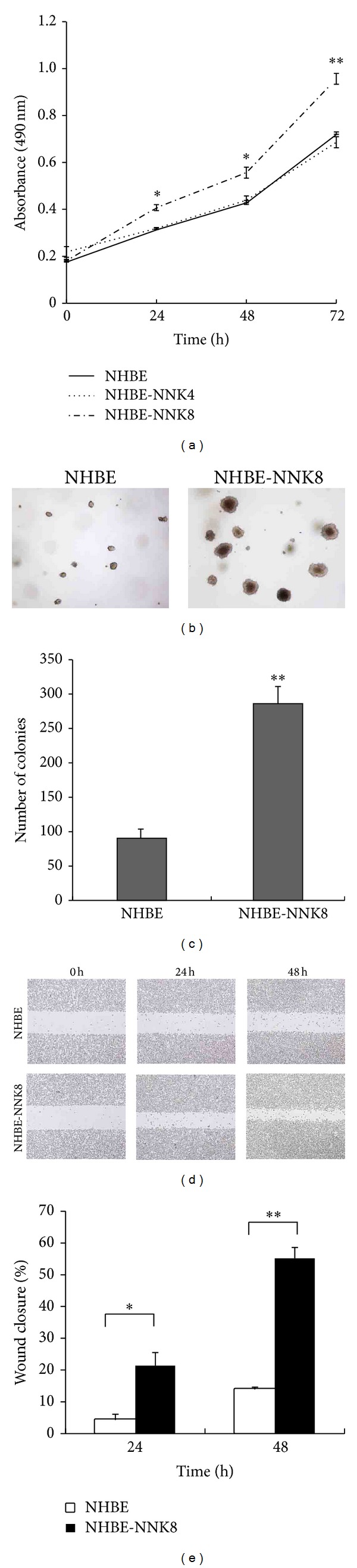
Malignant transformation of NHBE cells with NNK. (a) Eight weeks after NNK exposure, MTT assay was performed to measure cell proliferation rate. *P < 0.05, **P < 0.01, compared with NHBE cells. Values are the means ± SD of at least three independent experiments. (b) NHBE and NHBE-NNK8 cells were assayed for anchorage-independent growth in soft agar. Representative cell colonies are shown. (c) Quantitative analysis of colony numbers of (b). Values are the means ± SD of at least three independent experiments. **P < 0.01. (d) Analysis of cell migration in NHBE and NHBE-NNK8 cells by wound-healing assay. Representative photographs were taken at 0 h, 24 h, and 48 h after wound. Magnification, ×40. (e) The wound closure of (d) was quantified by measuring the remaining unmigrated area. Values are the means ± SD of at least three independent experiments. *P < 0.05, **P < 0.01.

After exposure to the tobacco-specific nitrosamine NNK for 4 cycles (NHBE-NNK4) and 8 cycles (NHBE-NNK8), NHBE cells were cultured in NNK-free fresh medium as described in methods. Eight weeks after exposure, we performed MTT assay to measure cell proliferation. NHBE-NNK8 cells showed increased proliferation rates, while NHBE-NNK4 cells showed no differences compared to the normal NHBE cells (Figure 1(a)). Then we conducted the anchorage-independent assay to determine the cell's ability to form colonies. More and larger colonies were found in soft agar with NHBE-NNK8 cells than the normal NHBE cells (Figures 1(b) and 1(c)). Furthermore, cellular mobility was evaluated using a wound-healing assay. NHBE-NNK8 cells exhibited a greater migration rate than the parental NHBE cells, as evidenced by the ability of NHBE-NNK8 cells to close more wound areas after 24 h and 48 h (Figures 1(d) and 1(e)). Our results indicate that NHBE-NNK8 cells have achieved the characteristics of malignant cells and confirm the malignant transformation ability of NNK.

### 3.2. Expression Changes of MMR Proteins after NNK Exposure and MSI

**Figure 2:**
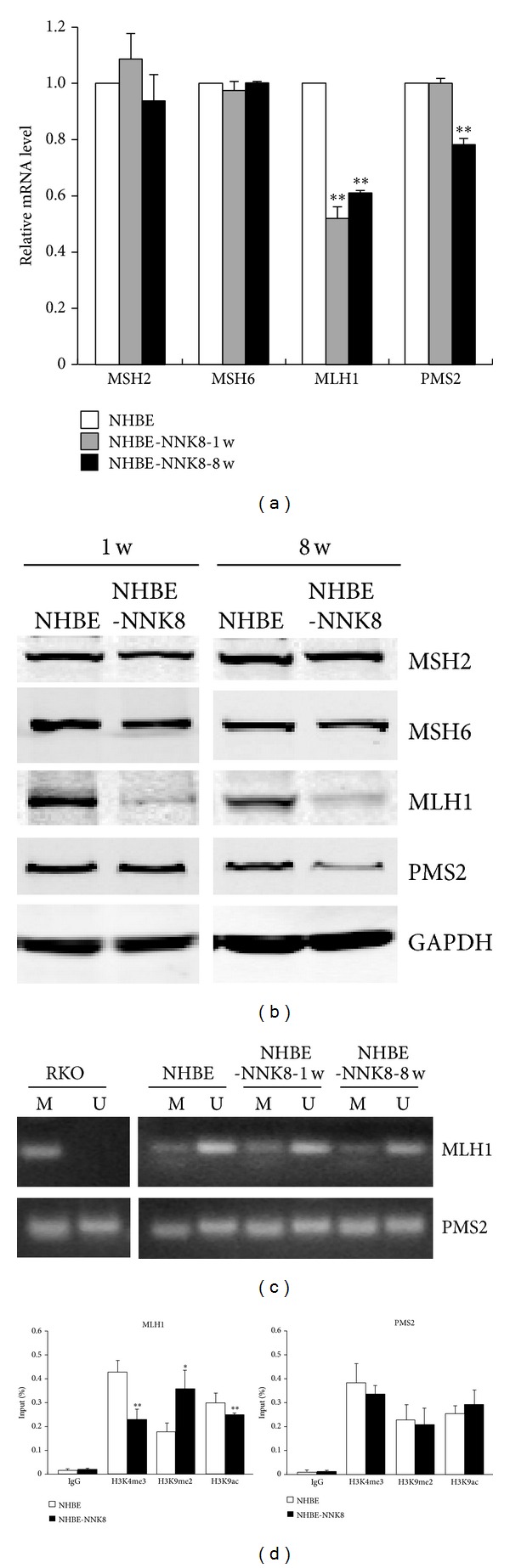
MMR proteins were downregulated after exposure to NNK. The mRNA (a) and protein (b) levels of MMR genes were detected at 1 week and 8 weeks after NNK exposure by real-time qPCR and Western blot. GAPDH was used as loading control. Values are the means ± SD of at least three independent experiments. **P < 0.01. (c) Results of* MLH1* and* PMS2* MSP assay using primers that amplify methylated (M) or unmethylated (U) alleles specifically. RKO cell was used as the positive control which has a hypermethylated level of* MLH1*. (d) ChIP assays were performed with indicated antibodies. The precipitated chromatin was quantified by quantitative PCR (qPCR) analysis. Data are presented as means ± SD from three independent experiments. *P < 0.05, **P < 0.01.

**Figure 3:**
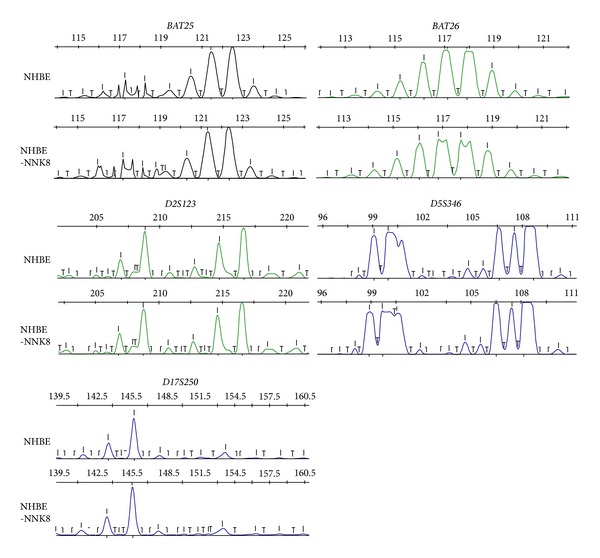
MSI analysis of NHBE and NHBE-NNK8 cells. A reference panel of five mono- and dinucleotide markers (*BAT-25*,* BAT-26*,* D2S123*,* D5S346*, and* D17S250*) was analyzed. No allelic shift in any of five microsatellite markers was observed.

To further investigate the mechanisms involved in NNK induced malignant transformation of NHBE cells, we detected the expression level of the DNA mismatch repair proteins, MSH2, MSH6, MLH1, and PMS2, in NHBE-NNK8 cells. One week after NNK exposure, only MLH1 showed significant downregulation in both the mRNA and the protein levels. However, 8 weeks after exposure, another MMR protein PMS2 also exhibited decreased expression level, while two other MMR proteins, MSH2 and MSH6, remained unchanged (Figures 2(a) and 2(b)). Since MLH1 was downregulated early and maintained in a low expression level in the NNK induced malignant transformation process, a possible involvement of epigenetic regulation was considered. Therefore, we analyzed the DNA methylation status of* MLH1* gene. Although the positive control RKO cells showed hypermethylated level of* MLH1*, both NHBE and NHBE-NNK8 cells exhibited an unmethylated* MLH1* DNA-dominant status with no significant differences (Figure 2(c)). Then, we profiled histone modification changes using ChIP analysis. The level of the “active” histone marks H3K9ac (acetylation at lys9) and H3K4me3 (trimethylation at lys4) decreased, whereas the “repressive” histone modification H3K9me2 (dimethylation at lys9) significantly increased at the promoter of* MLH1 *(Figure 2(d)). These data revealed that the downregulation of MLH1 is connected with the changed pattern of histone modification and is independent of DNA methylation. Similar analysis of DNA methylation and histone modifications was also performed in the PMS2 promoter region; no apparent changes were observed (Figures 2(c) and 2(d)). Meanwhile, NHBE-NNK8 cells were examined with a reference panel of five mono- and dinucleotide markers used for determining MSI. All five markers showed microsatellite stable (Figure 3).

### 3.3. Occurrence of p53 Mutation in NHBE-NNK8 Cells

**Figure 4:**
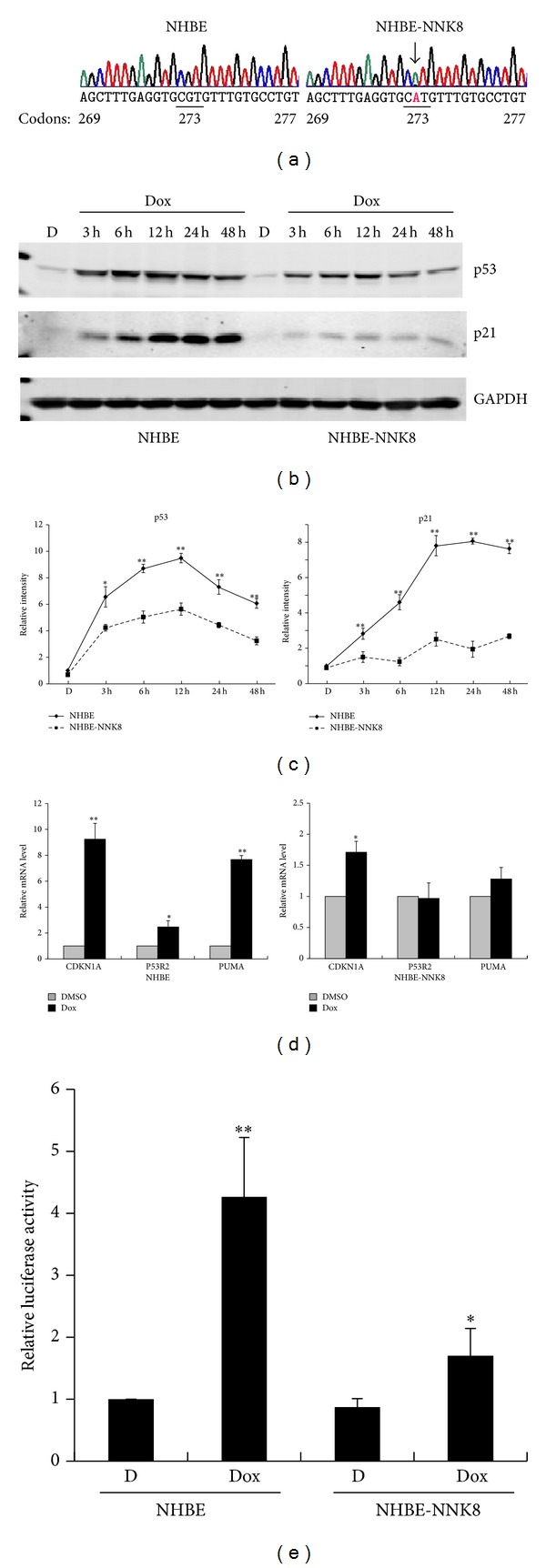
Occurrence of p53-R273H mutation in NHBE-NNK8 cells. (a)* TP53* mutation detected by directing sequencing in NHBE and NHBE-NNK8 cells. Mutation at codon 273 in exon 8 was shown in electropherograms. Base changed from CGT to CAT (black arrow). (b) NHBE and NHBE-NNK8 cells were treated with 1 *μ*M doxorubicin (Dox) or DMSO (d) vehicle control for the indicated times. p53 and p21 expression levels were assessed using Western blot analysis. GAPDH was used as loading control. (c) Quantitative data of (b) from three independent experiments (means ± SD). *P < 0.05, **P < 0.01, in comparison to NHBE cells treated with DMSO. (d) Quantitative RT-PCR analysis of p53 target genes,* CDKN1A*,* P53R2,* and* PUMA*, after treating NHBE and NHBE-NNK8 cells with 1 *μ*M doxorubicin (Dox) for 8 hours. (e) Luciferase activity analysis of* CDKN1A* promoter after 1 *μ*M doxorubicin (Dox) treatment. Luciferase activity is normalized for the transfection efficiency using cotransfection of pRL-SV40. Data are presented as means ± SD from three independent experiments. *P < 0.05, **P < 0.01, in comparison to NHBE cells treated with DMSO.

Given the central role of p53 in guarding the genomic integrity and its high frequency of mutation in human cancer, we analyzed the sequence of p53 exons 5–8 for mutations in the transformed NHBE-NNK8 cells. As shown in Figure 4(a), we identified a G>A mutation in* TP53* gene at codon 273 (R273H) in exon 8 in NHBE-NNK8 cells, and no mutation was found in the parental NHBE cells. The missense mutation R273H belongs to one of the hot-spot mutants found in p53. It is a DNA-contact mutant which resides in the DNA-binding domain of p53 and has compromised DNA-binding activity. So we analyzed the response of p53 with the treatment of doxorubicin, a DNA-damaging reagent known to activate p53. In NHBE cells, doxorubicin induced a significant elevation of p53 expression in a time-dependent manner, whereas a much weaker effect was detected in NHBE-NNK8 cells (Figures 4(b) and 4(c)). We then investigated the activation of p53 target genes after doxorubicin treatment. The cell cycle regulator p21 (*CDKN1A*) exhibited a dramatic upregulation in response to doxorubicin in both mRNA and protein levels in NHBE cells (Figures 4(b) and 4(c)). By contrast, the activation effect was weak in the transformed NHBE-NNK8 cells. Analysis of the promoter activity of p21 further demonstrated the activation differences between NHBE and NHBE-NNK8 cells (Figure 4(e)). Similar results were identified for other p53 target genes, such as DNA repair gene* P53R2* and apoptosis modulator* PUMA* (Figure 4(d)).

### 3.4. Restoration of p53 Function Inhibited the Proliferation of NHBE-NNK8 Cells

**Figure 5:**
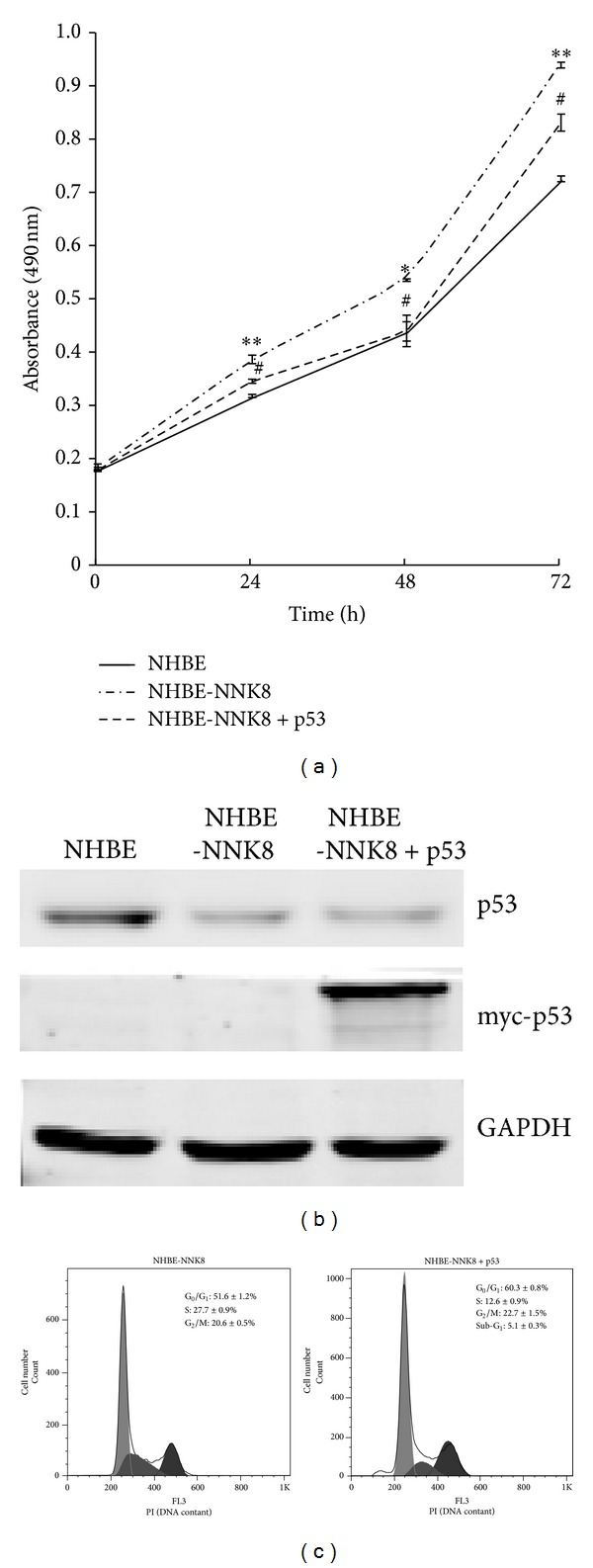
Restoration of wild-type p53 expression inhibited the proliferation of malignant transformed NHBE-NNK8 cells. (a) MTT assay was performed to measure cell proliferation rate. *P < 0.05, **P < 0.01, compared to NHBE-NNK8 and NHBE cells. ^#^ *P* < 0.05, compared to p53 transfected NHBE-NNK8 and NHBE-NNK8 cells. Values are the means ± SD of at least three independent experiments. (b) Western blot analysis of endogenous and ectopically expressed p53 in NHBE, NHBE-NNK8, and p53 transfected NHBE-NNK8 cells. GAPDH was detected as loading control. (c) DNA contents of NHBE-NNK8 and p53 transfected NHBE-NNK8 cells were analyzed by flow cytometry and FlowJo software. Numbers indicate the percentages of cells in the different phases of the cell cycle. Data are representative results from three independent experiments. PI, propidium iodide.

To study whether restoring the function of p53 can suppress the malignant proliferation of NHBE-NNK8 cells, we transiently transfected myc-tagged wild-type p53 in NHBE-NNK8 cells. The ectopically expressed p53 exhibited a normal activation response to DNA damage (data not shown). MTT assay showed that the proliferation rate was significantly decreased after reintroduction of wild-type p53 into cells (Figures 5(a) and 5(b)). The inhibition effect peaked at 48 h after transfection. Further analysis of cell cycle revealed that recovery of p53 function caused an increase in the percentage of cells in sub-G1 and G1 phase, suggesting that both cell cycle arrest and apoptosis could be activated in p53 transfected NHBE-NNK8 cells (Figure 5(c)).

## 4. Discussion

During the early stages of carcinogenesis, tumors might profit from the genetic instability and consequently genetic changes such as gene loss, gene amplification, point mutations, and chromosomal translocations. Genome instability is now considered as a characteristic of most cancers, especially hereditary cancers [22, 23]. In the present study, we investigated two important protectors of genome integrity, p53 and MMR system, in tobacco-specific carcinogen NNK-induced malignant transformation of human bronchial epithelial cells.

Human MMR system, including different functional complexes, plays a critical role in maintaining the fidelity of DNA replication by correcting mispaired nucleotides and insertion/deletion loops. In this system, MLH1 is one of the most frequently deficient components in human cancers, especially in gastrointestinal cancers [24, 25]. Interestingly, after exposure to carcinogen NNK, we also detected a significant reduction of MLH1 expression, emerging early and maintaining afterwards. The marked change in expression suggests an important role of MLH1 in NNK induced cellular malignant transformation. The tumor suppressor gene* MLH1 *is one of the most studied genes for epigenetic regulation in cancer. Its promoter methylation is directly involved in tumor progression, causing MMR dysfunction and consequent defect in repairing DNA replication errors [26, 27]. However, when we conducted the DNA methylation analysis of* MLH1* promoter, no obvious changes can be found between NHBE-NNK8 and the parental NHBE cells. It has been reported that histone modifications, such as H3K9me2, were associated with decreased* MLH1* mRNA expression [28–30]. Our further analysis also revealed that the “active” histone marks, such as H3K9ac and H3K4me3, were inhibited, whereas the “repressive” histone modification H3K9me2 was enriched in the* MLH1* promoter in NNK transformed cells. Therefore, a DNA methylation-independent but histone modification involved mechanism was employed in the silencing of* MLH1* gene during NNK induced malignant transformation.

We also found that another MMR protein PMS2 exhibited a slight but significant decrease in its expression level in a later stage of transformation. PMS2 can form the heterodimer MutL*α* with MLH1 to promote DNA repair [10, 31]. Its expression is frequently lost in MLH1 defect tumors and may facilitate the MSI and genomic instability [32, 33]. Although we did not observe apparent changes of DNA methylation and histone modifications in PMS2 promoter region, the long-term change of PMS2 and underlying mechanisms should be included in the future studies.

In contrast to colorectal cancer where the methylation or reduced expression of MMR genes is typically associated with high microsatellite instability (MSI-H), MSI is relatively rare in lung cancer, even in the presence of MMR deficiency, such as reduction or loss of MLH1 expression [34–36]. The mechanisms have remained largely unclear. When we assessed MSI of NNK transformed cells using a reference panel of five mono- and dinucleotide markers, no MSI phenotype was also detected. Beside the unknown mechanisms, one possibility of this phenomenon could be due to the remaining function of reduced but not totally lost expression of MLH1 and PMS2, and the other possibility could be insufficient time for developing MSI after NNK exposure. Further studies, such as mismatch repair assay and other types of microsatellite instability (elevated microsatellite alterations at selected tetranucleotide repeat, etc.), are required to elucidate potential mechanisms.

The* TP53* gene is frequently mutated in tobacco-related cancers. The mutations can be largely attributed to direct DNA damages induced by cigarette smoke carcinogens [7]. In this study, we also analyzed the mutation of p53 by direct sequencing. Intriguingly, a G>A mutation at codon 273 (R273H) was found in NNK transformed cells. The missense mutation R273H belongs to the most prominent mutation hot-spots in the* TP53* gene and alters the target DNA sequence [37]. It has been reported that the DNA-contact mutant R273H can exert dominant negative effects on the wild-type p53 protein by heterooligomerization and has compromised transactivity of downstream target genes, such as p21 (*CDKN1A*) [38–41]. When we investigated the response of p53 to DNA-damaging reagent doxorubicin, a significantly decreased induction of p53 expression and its transactivity toward downstream genes were found in NNK transformed cells, confirming the aberrant function of p53-R273H. Moreover, in addition to abrogating the tumor suppressor functions of wild-type* TP53*, R273H mutant has also been found to acquire new oncogenic activities to promote cancer, including metastasis promotion and increased resistance to anticancer treatments [42, 43].

To further evaluate the contribution of p53 to malignant transformation, we also restored the wild-type* TP53* expression by transient transfection in NHBE-NNK8 cells. The recovery of p53 expression significantly inhibited cell proliferation, suggesting a reversion of malignant properties. Previous reports have shown that reintroduction of p53 into cancer cells could induce a reversible G1 cell cycle arrest and G2 arrest, but not trigger apoptosis or cell death [44, 45]. We also analyzed the cell cycle after p53 restoration, and found an activation of G1 arrest. Additionally, a slightly but significantly increased apoptosis rate was detected in p53 transfected NHBE-NNK8 cells, indicating that both cell cycle arrest and apoptosis could be employed to inhibit the malignant cell growth by p53.

In summary, we reported the dysregulation of p53 and MMR system involved in carcinogen NNK-induced cellular malignant transformation. The early downregulation of MLH1 and a later incorporation of MSH2 defect indicate an important role of MMR in the initiation and progression of transformation. We also identified an R273H mutation of tumor suppressor p53 after chronic exposure to NNK. Occurrence of the mutation hindered DNA damage-induced activation of p53 and thus may contribute to the increased resistance to DNA damage in transformed cells. Finally, we demonstrated that recovery of p53 could inhibit the malignant proliferation through cell cycle arrest and apoptosis. These findings reveal that both genetic and epigenetic alterations of crucial genes are involved in tobacco carcinogen-induced transformation and provide evidence for developing novel therapeutic applications for tobacco-related cancers.
